# Supplementary material for: Normalized Raman Imaging for Studies of Tissue Physiology of the Kidney
Source: bioRxiv. 2025 Oct 14:2025.10.12.681746. Preprint. [Version 2] doi: 10.1101/2025.10.12.681746 (PMC12632984; doi:10.1101/2025.10.12.681746)
Supplement: Supplement 2 [file media-2.pdf]

Supplementary Table 1. Geometric features used by ResNet50 with descriptive overviews.

Feature	Description
Area	The total number of pixels enclosed within the contour.
Perimeter	The length of the contour boundary.
Aspect Ratio	The ratio of the contour's width to its height, as determined by its bounding box.
Extent	The ratio of the contour area to the area of its bounding box.
Solidity	The ratio of the contour area to the area of its convex hull, indicating how filled the contour is.
EquivDiameter	The diameter of a circle that has the same area as the contour.
Orientation	The angle at which the contour is oriented, typically calculated from an ellipse fitted around the contour.
Major Axis Length	The length of the longest axis of an ellipse fitted around the contour.
Minor Axis Length	The length of the shortest axis of an ellipse fitted around the contour.
Eccentricity	A measure of how elongated the contour is, calculated as the ratio of the distance between the ellipse's foci to the length of the major axis.
Convex Hull	The smallest convex shape that can fully enclose the contour.
Compactness	The ratio of the perimeter squared to the area, providing an indication of how compact the shape is.
Rectangularity	The ratio of the contour area to the area of its minimum enclosing rectangle.
Roundness (Circularity)	A measure of how closely the contour resembles a perfect circle.
Elongation	A measure of how stretched the contour is, often calculated as the ratio of the major axis length to the minor axis length.
Nuclei Number	The number of predicted nuclei inside the contour.
Minimal Distance and Average Distance	The average and minimal distance from the nuclei within the contour to the contour boundary, often used to assess nuclear distribution.
Minimal Distance and Average Distance between nuclei	The average and minimal distance between nuclei.
